# Whole Genome Sequencing of Mutation Accumulation Lines Reveals a Low Mutation Rate in the Social Amoeba *Dictyostelium discoideum*

**DOI:** 10.1371/journal.pone.0046759

**Published:** 2012-10-08

**Authors:** Gerda Saxer, Paul Havlak, Sara A. Fox, Michael A. Quance, Sharu Gupta, Yuriy Fofanov, Joan E. Strassmann, David C. Queller

**Affiliations:** 1 Department of Ecology and Evolutionary Biology, Rice University, Houston, Texas, United States of America; 2 Center for Biomedical and Environmental Genomics, University of Houston, Houston, Texas, United States of America; 3 Department of Biology, Washington University in St. Louis, St. Louis, Missouri, United States of America; UC Merced, School of Natural Sciences, United States of America

## Abstract

Spontaneous mutations play a central role in evolution. Despite their importance, mutation rates are some of the most elusive parameters to measure in evolutionary biology. The combination of mutation accumulation (MA) experiments and whole-genome sequencing now makes it possible to estimate mutation rates by directly observing new mutations at the molecular level across the whole genome. We performed an MA experiment with the social amoeba *Dictyostelium discoideum* and sequenced the genomes of three randomly chosen lines using high-throughput sequencing to estimate the spontaneous mutation rate in this model organism. The mitochondrial mutation rate of 6.76×10^−9^, with a Poisson confidence interval of 4.1×10^−9^ − 9.5×10^−9^, per nucleotide per generation is slightly lower than estimates for other taxa. The mutation rate estimate for the nuclear DNA of 2.9×10^−11^, with a Poisson confidence interval ranging from 7.4×10^−13^ to 1.6×10^−10^, is the lowest reported for any eukaryote. These results are consistent with low microsatellite mutation rates previously observed in *D. discoideum* and low levels of genetic variation observed in wild *D. discoideum* populations. In addition, *D. discoideum* has been shown to be quite resistant to DNA damage, which suggests an efficient DNA-repair mechanism that could be an adaptation to life in soil and frequent exposure to intracellular and extracellular mutagenic compounds. The social aspect of the life cycle of *D. discoideum* and a large portion of the genome under relaxed selection during vegetative growth could also select for a low mutation rate. This hypothesis is supported by a significantly lower mutation rate per cell division in multicellular eukaryotes compared with unicellular eukaryotes.

## Introduction

Mutations are the ultimate source of genetic variation upon which natural selection acts [1]. As such, mutations play a central role in the evolutionary process. How often new mutations arise has been difficult to determine until recently [2], [3]; mostly because mutations are very rare events [4]. In addition, many mutations have deleterious fitness effects [5], causing them to be quickly removed by natural selection. Therefore, unless methods are used to minimize selection, deleterious mutations can be undercounted. Estimates based on comparative approaches are further hampered by unknown times of divergence and unknown selection pressures imposed by environmental variation during divergence. Until recently, direct estimation of mutation rates was mostly limited to the analyses of a few genes based on phenotypic assays [4].

A new and promising approach to studying mutation rates is the combination of mutation accumulation (MA) experiments with whole-genome sequencing using high-throughput technologies [6], [7], [8], [9], [10], [11], [12], [13], [14]. The advent of cost-effective sequencing now makes it possible to detect mutations such as substitutions, deletions, insertions, and gene duplications directly at the molecular level in both coding and non-coding regions of the genome [3]. MA experiments have a number of advantages over other methods for studying mutation rates. These types of experiments allow spontaneous mutations to accumulate regardless of their effects on fitness, as long as they are not severely deleterious. Natural selection can be relaxed by repeatedly reducing the population size to one individual in asexually reproducing organisms, or to a few closely related individuals (often siblings) in sexually reproducing organisms. This process prevents deleterious (but not fatal) mutations from being eliminated by competition and allows them to be as likely to be fixed by drift as other alleles. Replicated populations sharing a single common ancestor can be propagated under identical experimental conditions for a known number of generations and allowed to accumulate independent, random mutations. These results can be compared among species, with the advantage of matching methodologies across very different life cycles.

Several estimates of eukaryotic, spontaneous, nuclear mutation rates obtained from the whole-genome sequencing of MA lines have been published. Of these, the estimated mutation rate of *Arabidopsis thaliana* (7×10^−9^) [10] is the highest. The lowest rate was estimated in *Saccharomyces cerevisiae*: 3.3×10^−10^ for asexual haploid cells [9], 2.9×10^−10^ for asexual diploid cells dividing mitotically, and 3.9×10^−10^ for diploid cells with recombination [15]. *Drosophila melanogaster* (3.5×10^−9^) [13] and *Caenorhabditis elegans* (2.7×10^−9^) [6] are estimated to have intermediate mutation rates.

Current mutation rate estimates for the mitochondrial genome from MA experiments suggest a relatively constant mutation rate across different organisms. The highest mitochondrial mutation rate has been estimated in *Daphnia pulex* at 1.37×10^−7^ for sexual lines and 1.73×10^−7^ for asexual lines [16], while the lowest mitochondrial mutation rate was estimated for haploid asexually reproducing yeast at 1.29×10^−8^ [9]. The estimated mitochondrial mutation rates for *C. elegans* and *D. melanogaster* are intermediate at 9.7×10^−8^ and 6.2×10^−8^, respectively [7], [17].

**Figure 1:**
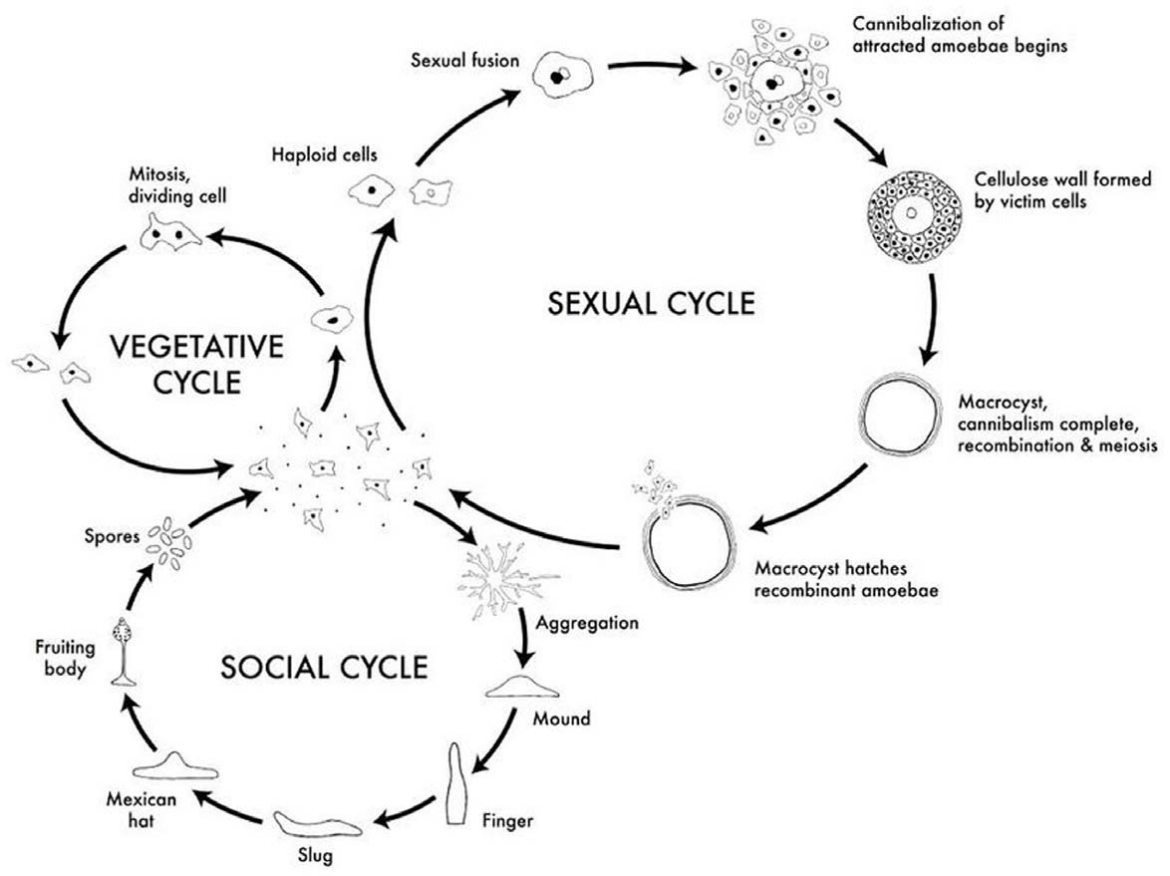
Life cycle of *D. discoideum* showing the vegetative, social, and sexual. During the vegetative cycle, single cells feed on bacteria and divide mitotically. Upon starvation, single cells aggregate and, depending on environmental conditions, enter the social cycle or the sexual stage. During the social stage, single cells aggregate and form a multicellular fruiting body that consists of a stalk made of dead cells holding aloft a sorus containing the fertile spores. During the sexual stage, two haploid cells fuse and ingest the other aggregating cells to form a macrocyst. This figure was published under CC Creative Commons Attribution - Share Alike 3.0, David Brown & Joan E. Strassmann and is available at http://www.dictybase.org/Multimedia/DdLifeCycles/index.html.

We combined an MA experiment and whole-genome sequencing to estimate the spontaneous single nucleotide mutation rate in the social amoeba *Dictyostelium discoideum.* This haploid eukaryote is a model system for the evolution of sociality [18], [19], [20], multicellularity, developmental and cellular biology [21], [22], and pathogenicity [23], [24], [25]. The social amoeba has a complex life cycle with a vegetative unicellular stage, a social multicellular stage, and a social sexual stage (Figure 1). During the vegetative stage, single cells live in soil and prey on microorganisms. Upon starvation, single cells start to aggregate, and then, depending on environmental conditions, enter either the social or the sexual stage. During the social, multicellular stage, single cells aggregate and undergo complex behavior that culminates in the formation of a fruiting body, which consists of fertile spores contained within a sorus held aloft by a stalk made of dead cells [26], [27], [28]. During fruiting body formation, about 20% of the cells die to form the stalk. Unlike other multicellular organisms that go through a single-cell bottleneck where all cells are essentially clones of the initial zygote, the aggregating cells in *D. discoideum* can be genetically different. This can lead to conflict over spore and stalk allocation [20]. An alternative to the social cycle is the sexual cycle. During the sexual cycle, two cells of different mating types fuse and form a giant cell that cannibalizes all other aggregating cells, forming a macrocyst that can become dormant and survive harsh environmental conditions [26]. This complex life cycle makes it difficult to clearly specify generations, and we have previously called one vegetative cycle combined with fruiting-body formation a social generation [29]. During our MA experiment, there is no fruiting stage or sexual stage; the cells remained in the single-cell stage. Therefore, we refer to a single replication event that manifests itself in the division of a single cell into two daughter cells as one generation.

The haploid nuclear genome of *D. discoideum* is 34 MB and contains six chromosomes [30]. As of September 11, 2012, 12,646 protein-coding genes have been identified, and 188 pseudogenes have been annotated on dictybase (http://www.dictybase.org). The genome is very AT-rich (78%), and contains over 11% simple-sequence repeats [30]. Roughly 2/3 of the genome is coding sequence. The mitochondrial genome contains 55,564 bases, is slightly less AT-rich than the nuclear genome (72.57%), and encodes 41 genes. On average, one *D. discoideum* cell contains about 200 copies of the mitochondrial genome [26].

Several lines of evidence suggest a relatively low mutation rate for *D. discoideum*. Analysis of genetic variation in wild populations indicated low levels of genetic variation, which could be explained by low mutation rates [31]. Similarly, *D. discoideum* is very resistant to DNA-damaging agents, which suggests efficient DNA-repair mechanisms [26], [32], [33]. The most direct evidence for low mutation rates comes from the analysis of microsatellite mutations in the same 90 MA lines used here; *D. discoideum* has the lowest per-generation per-repeat mutation rate reported [34].

We randomly selected three of the same MA lines used to estimate microsatellite mutations (MA31, MA47, and MA55) and sequenced their whole genomes to determine if the genome-wide point mutation rate is also low; or alternatively, if the low mutation rate is a characteristic of the microsatellite regions only.

## Results

We sequenced the whole genomes of three lines that had accumulated mutations through 70 single-cell bottlenecks, which equals 1000 cell divisions. On average, we aligned 21.5 million reads from each MA line to the sequenced reference genome [30]. To maintain high sequencing quality, we trimmed all the reads to 31 bp, regardless of the actual read length. The average read coverage per site in the nuclear genome ranged from 9X to 11X (lines MA55 and MA31 respectively), while the average coverage in the considerably smaller and more abundant mitochondrial genome was close to 5000X (4655X for MA31, 7141X for MA47, and 3033X for MA55).

### Nuclear Genome Mutation Rate

Mutations are very rare events, so it is essential to use stringent and uniform procedures to identify true mutations above the background noise of sequencing and alignment errors. High levels of read coverage tend to increase the confidence for a base call. Increasing the minimum coverage required for individual base calls, however, may come at a cost of reduced information overall, because a smaller percentage of the genome will be covered at the minimum level. To reduce the errors and retain as much of the genome as possible, we used a minimum coverage of five reads, which is half the average coverage. We required at least 90% agreement among the reads mapped to a given site in the reference genome before we called the base at that site in an MA line.

To reduce alignment errors caused by repetitive or very similar sequences, we excluded sites with greater than three times the average coverage and also those that were not uniquely mappable (see Methods). We determined that 59% (20,337,176 bases) of the nuclear genome could be mapped uniquely with 31-bp reads. The AT content of the bases passing our filters was 75%, which is slightly lower than the AT content of the nuclear genome (78%), probably because of the exclusion of highly AT-rich repetitive regions that could not be mapped accurately [30].

**Figure 2:**
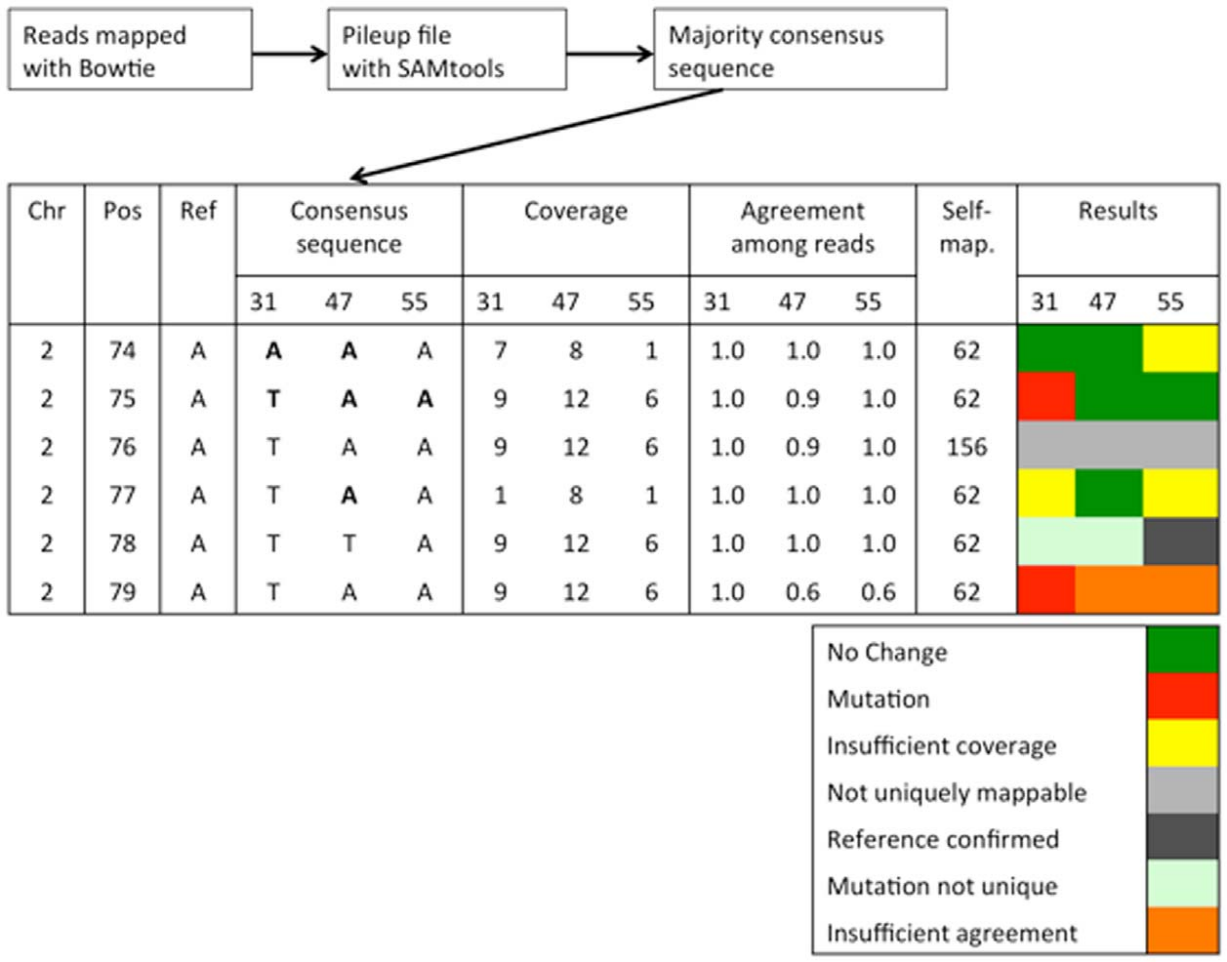
Identification of unchanged sites and unique mutations. Analysis pipeline and examples of rules used to identify mutations (red) unique to one mutation accumulation line, and unchanged nucleotides (green). Each line represents a hypothetical position in the genome and is characterized by the chromosome (Chr), position (Pos), and the reference base (Ref). Columns 4–6 list the majority consensus base for the three MA lines (MA31, MA47, MA55). Columns 7–9 show the number of reads covering this particular position in the three MA lines, columns 10–12 list the fraction of reads agreeing with the majority base. We used a minimum agreement of 90%. Column 13 gives the read coverage for this position for the self-mapping of the genome and indicates whether the position is uniquely mappable (if self-mapping coverage = 62). Columns 14–16 give the results from our filtering criteria, with unchanged sites indicated in green, mutations in red, and confirming bases in yellow. Positions that were not covered in all three lines were excluded from the analysis.

We eliminated candidate mutations that showed evidence of existing in multiple lines because these were likely due to errors in the reference genome, differences between our ancestor clone and the reference genome, or mutations that occurred while we were growing up cells to start our 90 lines. Although we required a minimum coverage of five with 90% agreement among reads to identify candidate mutations, we used weaker criteria – minimum coverage of one with 50% agreement – when seeking evidence of the same mutation in other lines. This was conservative; we did not want to accept a candidate mutation if there was any reasonable evidence that it was not a true mutation. In order to prevent this procedure from introducing a bias into our rate estimates, we applied a parallel rule for sites determined to be unchanged: a minimum coverage of five and 90% agreement among the reads to identify candidate sites, and confirmation at a minimum coverage of one and 50% agreement by the other lines (Figure 2 illustrates these procedures).

Using the approach described above, we identified a total of 34,653,717 unchanged bases meeting our criteria in the three genomes, about the size of one complete nuclear genome. Overall, we identified 1917 candidate mutations compared with the reference genome, but 1916 of them were ruled out because they appeared in more than one line. Only one of these candidate mutations was a true mutation unique to a single line. Based on the identified mutation and unchanged nucleotides, we estimated the mutation rate to be: 1 mutation/(34,653,718 possible sites×1000 generations) = 2.9×10^−11^ per site per cell-generation. The mutation that we observed was an A to T substitution in a noncoding region on chromosome 2, position 928240, in line MA31. We confirmed that this mutation was unique to MA31with Sanger sequencing. The Poisson confidence interval for the number of mutations ranged from 0.0255 to 5.572, which results in a Poisson confidence interval for the mutation rate ranging from 7.4×10^−13^ to 1.6×10^−10^ mutations per site per cell-generation.

### Sequencing and Alignment Error Rates

To assess the error rates in sequencing and alignment, we calculated the fraction of reads that did not agree with the majority base call at their site, using an approach similar to that of Keightley et al. [13]. If we calculate the error rate for all aligned reads without applying our filtering criteria, we get an overall average error rate of 2.3×10^−3^ (95% CI: 2.4×10^−3^, n = 3) per base read for all the aligned reads. The error rate decreases to 1.3×10^−3^ (95% CI: ±1.5×10^−3^, n = 3) if we only consider sites that fulfill our selection criteria. These error rates are comparable to those observed by Keightley et al. [13]. We considered not trimming our reads to 31 bases and using 36 bases instead, but did not do so because the estimated error rate for the extra five bases was close to an order of magnitude higher (0.0127).

### Mitochondrial Mutation Rate

We estimated the mitochondrial mutation rate in roughly the same manner as the nuclear rate, but with some adjustments for heteroplasmy. For sites with mixed reads, we needed to select a frequency cutoff to separate low-frequency heteroplasmic mutations from sequencing errors. We chose a cutoff of 0.03, because two different methods suggested that we could distinguish errors from mutations at that frequency (see Materials and Methods). If the frequency of a mutant allele was equal to or exceeded 3%, it was counted as a mutated heteroplasmic site rather than one with sequencing errors. To estimate the mitochondrial mutation rate, we assumed that mutations are rare and the fate of a mutation is determined by drift. The same procedure was used previously by Haag-Liautard et al. [7]. Therefore, the probability of fixation of a mutation at the drift-mutation equilibrium is equal to the frequency of the mutation, and the heteroplasmic sites contribute to the mutation rate estimate for one MA line in proportion to their frequency as the (sum of f(m))/(n*g), where *f(m)* is the frequency of a unique mutation, *n* is the number of unchanged nucleotides and *g* is the number of generations. Using our cutoff of 0.03, we estimate the mitochondrial mutation rate to be 6.76×10^−9^ per nucleotide per generation (Poisson confidence interval: 4.1×10^−9^ − 9.5×10^−9^). We identified a total of 19 mitochondrial mutations (six in MA31 and 12 in MA47, both with 97% coverage of the genome; and one in MA55 with 91% coverage). Most mutations occurred at a frequency lower than 0.1. Only one mutation in MA47 reached a frequency of 0.44. We did observe candidate mutations that occurred at higher frequencies, but none of them was unique to one MA line. Fortunately, the choice of the cutoff threshold had relatively little effect on the estimate: for a cutoff of 0.01, the mutation rate estimate was 1.44×10^−8^; and for a cutoff of 0.10, it was 1.11×10^−8^.

### Alternate Alignments and Modified Filtering Parameters

Different mapping tools, methods to compile a consensus sequence, and filtering techniques all consistently identified the same single nuclear mutation described above. The maximum likelihood (ML) method developed by Lynch [9], [35] was unable to estimate a mutation rate, because it exhausted all possible mutations within two iterations. Changing our filtering parameters had little effect on the estimated mutation rates. Lowering the minimum coverage or increasing the maximum coverage did not increase the number of unique mutations; but instead increased the number of unchanged nucleotides, leading to slightly lower mutation rate estimates. The opposite trend was observed when we required that 100% (instead of 90%) of the bases agree with the majority base call. Increasing the minimum-agreement requirement did not alter the number of mutations identified; it did, however, reduce the number of unchanged nucleotides and thus increase the mutation rate, albeit very slightly (from 2.89×10^−11^ to 2.97×10^−11^).

## Discussion

We combined an MA experiment and whole-genome sequencing and estimated the single-nucleotide mutation rates for mitochondrial and nuclear DNA in the social amoeba *D. discoideum*. The mitochondrial mutation rate of 6.76×10^−9^ (with a Poisson confidence interval ranging from 4.1×10^−9^ to 9.5×10^−9^) per nucleotide per cell-generation is based on 19 unique mutations in the three genomes and is lower than the mitochondrial mutation rates observed in other species, which range from 1.29×10^−8^ in *S. cerevisiae* [9] to 17.3×10^−8^ in the asexually reproducing *D. pulex* [36] with *C. elegans* and *D. melanogaster* falling in between [7], [17].

We estimated the nuclear mutation rate to be 2.89×10^−11^ per site per generation with a Poisson confidence interval ranging from 7.4×10^−13^ to 1.6×10^−10^. This estimate is based on one single nucleotide mutation within three experimental lines. Given this per-site mutation rate and the genome size and number of generations, we would expect there to be one mutation on average in each experimental genome over the 1000 generations.

**Figure 3:**
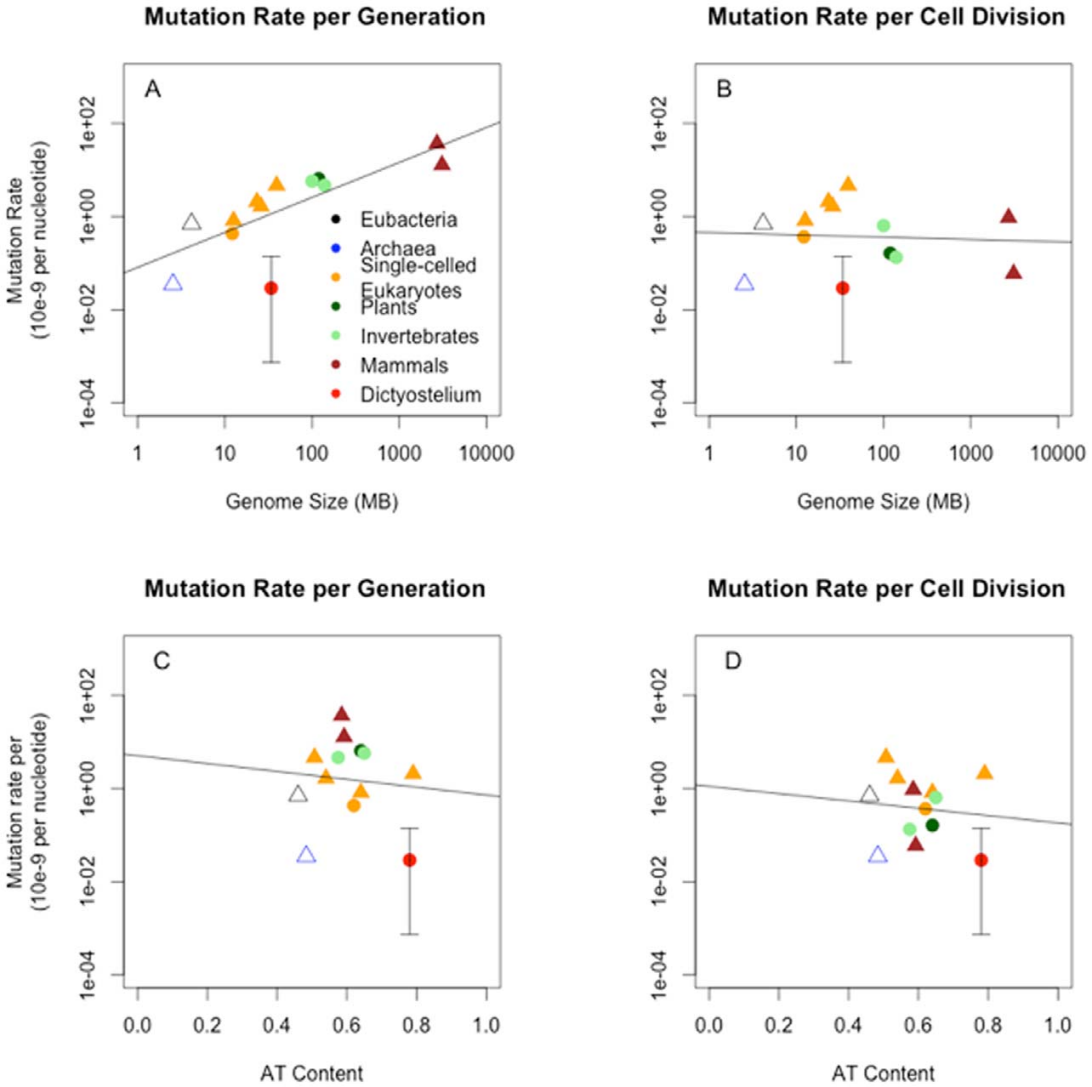
The relationship of mutation rates and genome size or AT content. Mutation rate (as X×10^−9^ per nucleotide) per generation and mutation rate per cell division are plotted as a function of genome size (in MB) (A and B) and as a function of AT content (C and D). We observed a significant relationship between mutation rate and genome size (Log_10_(mutation rate) = −1.0909+0.7505×log_10_(Genome size), with R^2^ = 0.58 and p = 0.0024.), but not for mutation rate per cell division or mutation rates and AT content. *D. discoideum* is represented by the red dot; the error bars indicate the Poisson confidence interval for our nuclear mutation rate. Mutation rates for Eubacteria and Archaea are given as averages of multiple estimates and are represented by open symbols. The average mutation rate of Eubacteria does not include *Buchnera aphidicola* due to its unusually high mutation rate, which is characteristic for endosymbionts. Circles represent mutation rate estimates obtained from high-throughput sequencing of MA lines; estimates obtained through other methods are represented by triangles. Mutation rates for yeast are calculated as the average from Lynch [8] and Nishant et al. [15]; all other estimates are from Lynch [8].

Our estimate for the nuclear mutation rate is the lowest spontaneous mutation rate reported to date for a eukaryote [8] (Figure 3). Because there was only one mutation, the confidence interval of our estimate spans two orders of magnitude; but even so, the upper limit is still considerably lower than any other per-generation mutation rate for eukaryotes [8].

Combining our new estimate with previous estimates for other cellular organisms used by Lynch [8] shows that mutation rate increases significantly with genome size (Figure 3A); though if we restrict our analysis to the five estimates obtained by whole-genome sequencing, the regression loses significance. To compare mutation rates across taxa, we scaled all of the estimates to the number of cell divisions instead of generations by using the numbers of germ-line divisions per generation published in Lynch [8]. After accounting for the number of germ-line cell divisions per generation, we no longer observe a significant effect of genome size on mutation rates. While *D. discoideum* still has the lowest point estimate, the confidence interval includes the mutation rate estimates for plants, *D. melanogaster* and *Homo sapiens*, while all other single-celled eukaryotes have considerably higher mutation rates (Figure 3B).

Given our very low nuclear mutation rate estimate, we focus on three questions. First, are there any biases that might result in an estimate that is far lower than the true value? Second, are there any other data supporting a low mutation rate in *D. discoideum*? Finally, are there reasons why we might expect the mutation rate to be low in this species?

### Possible Biases

Several factors could potentially lead to an underestimation of the mutation rate in *D. discoideum*. The high AT content combined with an unusual abundance of repeat sequences and similar short sequences throughout the genome make it impossible to align many short reads without ambiguity. The exclusion of such sites could downwardly bias our estimate if point mutations are more likely in the repeat regions. It is likely, however, that estimates in other species would incorporate a similar bias, and we therefore consider this an unlikely explanation for *D. discoideum* having a lower rate.

Our estimate is based on strains that evolved during an MA experiment in the laboratory under controlled, benign conditions. The cells were never exposed to UV light, were fed non-pathogenic bacteria, and grew on rich nutrient agar. Environmental factors such as UV radiation and DNA-damaging compounds in soil and the bacteria eaten by *D. discoideum* could lead to more mutations in the wild. MA experiments for other species, however, were also performed under benign laboratory conditions. Nevertheless, if *D. discoideum*’s natural environment is more mutagenic than those of other species, it is possible that its mutation rate in the wild is less different from those of other species than the estimates suggest.

The low mutation rate we observed in our three lines raises the question of potential cross contamination of the lines during the MA experiment, but multiple lines of evidence suggest this is very unlikely. As outlined in the Material and Methods, we used extra precautions to prevent any cross contamination and mixing up of the lines. The three strains we used are distinguishable by microsatellite mutations [34]. Mixing up the lines during the DNA extraction and library prep is not consistent with our finding of multiple mitochondrial mutations that were unique to one MA line only. Our mutation rate for the mitochondria is very similar to previous estimates for the mitochondrial mutation rates. Finally, even in the extreme case of two of our lines actually being the same line (a possibility excluded by the above evidence), the number of mutable sites would be reduced by 1/3, and the estimated mutation rate would increase by only 50%.

Finally, we should consider the possibility that our analysis was too conservative and led to an artificially low mutation rate compared with other studies. This seems unlikely for two reasons. First, our approach was very similar to the approach used to estimate the mutation rate of *D. melanogaster*. Second, mutation rate estimates are much more sensitive to the number of mutations than to the number of unchanged sites. Most of the filtering criteria we used were identical to the ones used by Keightley et al. [13] (three lines, same average coverage, minimum coverage of five, 90% agreement among reads, and coverage of all three lines at a site), with the exception that we only considered uniquely mappable sites and relaxed the coverage requirement in confirming MA lines. Using only uniquely mappable sites will miss some reads at sites where some but not all reads can be mapped; but because the same criterion was applied to changed and unchanged sites, it should not affect our estimates. Relaxing the coverage of confirming MA lines allowed us to consider more sites (33% of the genome). Requiring a minimum coverage of five for all lines at a given site reduced the fraction of the genome under consideration to 21%, and only slightly increased the mutation rate to 4.4×10^−11^ with a Poisson confidence interval ranging from 1.4×10^−12^ to 2.5×10^−10^, which is still considerably lower than other eukaryotic mutation rate estimates. The relatively small effect of changing the minimum coverage requirement also suggests that our relaxed comparison-coverage does not account for the 100-fold difference in mutation rates between *D. melanogaster* and *D. discoideum*. While even smaller fractions of the genome have been used to estimate mutation rates (e.g., roughly 15% was used for *C. elegans* [37]), we decided to use as much of the genome as possible by applying the relaxed coverage requirements for the confirming bases.

The mutation rates for *S. cerevisiae* [9] and *C. elegans* [37] are both based on ML methods. We used the ML method developed by Lynch, but were unable to estimate the mutation rate with only one mutation. The ML method correctly identified the one mutation, but failed to estimate a mutation rate. Regardless of the method used to estimate mutation rates, the mutation rate estimates are much more sensitive to changes in the number of mutations than they are to changes in the number of unchanged nucleotides. We were very carful to use the same criteria to identify mutations and unchanged nucleotides, in order to avoid overestimating one or the other. The results of using different approaches, methods, and parameters suggest that the mutation rate estimate is very robust to changes in the analysis, as we were only able to detect one mutation overall regardless of changes in the analysis. No other approach or method was able to identify more mutations that could be confirmed with Sanger sequencing.

### Other Data on Mutation Rate

Our low mutation rate estimate is consistent with low levels of genetic variation observed in *D. discoideum* strains isolated from the wild across a large geographic range [31]. Our estimate is also consistent with the unusually low microsatellite mutation rates for both dinucleotide and trinucleotide repeats across all 90 lines in our MA experiment [34]. The average microsatellite mutation rate was 1.1×10^−7^ per repeat per generation, which is lower than the average microsatellite mutation rates observed in *S. cerevisiae* [9], *D. melanogaster* [38], *C. elegans*, and *D. pulex* [36].

On the other hand, the 90 lines used in our MA experiment did show the expected kinds of effects on fitness-related phenotypes including growth rates, slug migration, and fruiting body characteristics [39]. In general, the means declined and the variances increased. Numerical estimates of the phenotypic mutation rates will be reported elsewhere, but the data certainly indicated that there was more than one mutation per genome over the course of the experiment. This suggests that many phenotypic changes were caused by additional mutations that were either in regions that we could not assess (such as repetitive regions) or caused by non-point mutations (including microsatellite repeat-number changes, as well as other insertions and deletions).

Our low mutation rate is also consistent with the reported high resistance in *D. discoideum* to DNA-damaging agents, which suggests efficient DNA-repair mechanisms [26], [32], [33], [40]. Efficient DNA repair is a likely adaptation to a soil dwelling and predatory life style, during which the amoebae come in contact with, and often engulf, microorganisms that secrete DNA-damaging compounds [26].

### Reasons for Low Mutation Rates

If *D. discoideum*’s natural environment is indeed unusually mutagenic, then in order to keep its natural mutation rate at levels appropriate for its genome size and effective population size, it would likely have evolved particularly efficient DNA-repair mechanisms. Then, as noted above, its laboratory mutation rate in the absence of mutagenic agents would be unusually low, essentially as a side-effect.

The very high AT content of *D. discoideum* might also lower its mutation rate. A commonly observed mutational bias in various taxa is that G/C to A/T transversions occur at a higher rate than A/T to G/C transversions [37], [41], [42]. Genomes with high AT content have fewer G and C sites with high mutation rates, and more A and T sites with low mutation rates, and should therefore also have lower mutation rates overall. However, the expected effect may not be large. If the AT content of *D. discoideum* were at mutational equilibrium, with the A/T to G/C rate at *m*, then the G/C to A/T rate would be 3 *m*. Therefore an AT bias in the genome would have a relatively minor effect on the overall mutation rate (compared to a 50% AT genome with average rate ½ *m* + ½ 3 *m* = 2 *m*, it would have a mutation rate only 25% lower: ¾ *m* + ¼ 3 *m* = 1.5 *m*). The difference could be larger if AT/GC percentage is determined partly by selection. Using the comparative data shown in Figure 3C, we observed a negative, but not significant, relationship between AT content and mutation rate per generation. This relationship changed little whether the mutation rate was per generation or per cell division (Figure 3D). A multiple regression that included genome size and AT content did not show a significant effect of AT content on mutation rate per generation or per cell division.

The unusually low mutation rate in *D. discoideum* could also be a result of its unusual social life cycle. On occasion, starved *D. discoideum* cells aggregate to form a multicellular fruiting body with asexual spores. The genes involved in the multicellular stage comprise about 25% of the genes in *D. discoideum* and undergo a dramatic transition from no expression to high expression over the course of fruiting [43]. This is in contrast with yeast, for example, where only 6% of the genes seem to be involved in sporulation [44]. During the course of our MA experiment, the cells were propagated as single cells in vegetative growth with no fruiting. Although their fruiting phenotypes changed, all 90 MA lines maintained the ability to form fruiting bodies throughout the experiment [39], [45], suggesting that none of them acquired strongly deleterious mutations in genes involved in aggregation, fruiting body formation, and spore production. This result is quite remarkable considering that selection was eliminated not just by bottlenecking, but also by the fact that these traits were never expressed during the experiment. In its natural environment, *D. discoideum* must also pass through many cell generations – precisely how many is not known – between fruiting episodes, so the many genes that are primarily expressed during the multicellular stage [43] could accumulate mutations without selective purging. A low mutation rate could be an adaptation to reduce the accumulation of deleterious mutations in developmental genes during prolonged vegetative growth, and thus ensure the important ability to form fruiting bodies and spores. This hypothesis does not predict a similarly low mutation rate in the mitochondrial genes, and the finding that the *D. discoideum* mitochondrial mutation rate is not especially low fits.

If the hypothesis that the low genomic mutation rate in *D. discoideum* is, at least in part, an adaptive mechanism to maintain essential life-history traits that are only periodically expressed is valid, then it should also apply to other multicellular organisms. For example, a tree produces complex and important flowers only after a very long series of cell divisions in which no flowers are produced; mutations could thus accumulate in the flower-specific genes. So why is the *D. discoideum* mutation rate so low compared with multicellular plants and animals (Figure 3A)? Part of the answer is that MA experiments on multicellular organisms estimate mutation rates per multicellular generation whereas we necessarily estimated the mutation rate for *D. discoideum* per cell division, because we do not know how many cell divisions make up a multicellular generation in nature. If there are 10–100 cell divisions between *D. discoideum* fruiting cycles, then the mutation rate per multicellular generation would be 10–100 times higher than our estimate, and plotting that point in Figure 3A would place *D. discoideum* much closer to the regression line. Another way to illustrate this point is to calculate all mutation rates per cell division (Figure 3B). In that case, the *D. discoideum* estimate is still at the low end, but is not an extreme outlier. Indeed, there appears to be some support for the idea that multicellular eukaryotes have lower mutation rates per cell division than unicellular ones; this is consistent with the hypothesis that they need to be lower to maintain important multicellular traits that are not expressed in most cells. A Wilcoxon rank test of mutation rates per cell division between unicellular and multicellular eukaryotes was marginally significant when we excluded *D. discoideum* from the analysis (W = 3, p = 0.056). Including *D. discoideum* as a multicellular eukaryote, as it is with respect to the possession of a large set of stage-specific genes that are expressed only rarely, resulted in a significant difference between the per cell division mutation rates of unicellular and multicellular eukaryotes (W = 3 and p = 0.03).

Our estimate of the spontaneous genomic mutation rate in *D. discoideum* of 2.9×10^−11^ per site per cell division is currently the lowest estimate for a eukaryote and may raise the question of how low can mutation rates be. This mutation rate is close to the most recent mutation rate estimate in *E. coli*, which was estimated to be 8.9×10^−11^ per site per generation [46]. The cost of replication fidelity is expected to impose a lower limit on mutation rates [47]. With an increased number of mutation rate estimates in the future, we will undoubtedly be able to get a better estimate for the lower limit of mutation rates and be able to gain a better understanding of one of the most elusive parameters in evolutionary biology.

## Materials and Methods

### Mutation Accumulation Experiment

We created 90 MA lines and 10 control lines by starting each line from a single clone of the ancestor AX4, as described in McConnell et al. [34]. We propagated each line independently for 140 days by reducing the population size to a single cell every other day, resulting in 70 single-cell bottlenecks and 1000 vegetative divisions or generations (with no fruiting body formation). We grew the cells at low density on a fresh lawn of bacteria. After two days, plaques were visible, allowing us to pick up the cells of a randomly chosen plaque and streak them onto a fresh lawn of bacteria. Cells in a distinct, round plaque all originated from a single cell. We repeated this process every other day. In order to reduce the potential for cross contamination among the evolving lines, we used several precautions. We transferred the 100 lines in batches of ten (i.e., 1–10, 11–20, and so forth). The three lines that we sequenced were in separate batches and hence very unlikely to have been mixed (note that mixing within a batch, or with any non-sequenced line, would not bias the estimated mutation rate – it would simply substitute one line for another). The transfers were done by at least two people, who both checked the identity of the line to be transferred and the new plate identity. If there was any question about the identity of a line, the plates were discarded and we repeated the questionable transfer using backup plates. Every ten bottlenecks, we froze a sample of the population. To freeze the populations, we grew each line to high density, collected the spores after fruiting body formation, and froze each line along with the ancestor at −80°C in KK2 buffer (14.0 mM KH_2_PO_4_ and 3.4 mM K_2_HPO_4_, pH = 6.4) supplemented with 20% glycerol. While all lines remained in vegetative growth during the MA experiment, they retained the ability to form fruiting bodies throughout the experiment.

### Number of Cell Generations

We estimated the number of cell divisions during the 48-hour period between transfers by counting the number of cells in each of 16 48-hour plaques of the ancestral clone and taking the logarithm with base two. This yielded an estimate of 14.22 divisions (± st.dev. 0.544 divisions). The rate was also estimated for the 90 MA lines, and did not differ [39]. Multiplying by 70 transfers yields 995 cell divisions, slightly lower than the 1007 divisions reported previously [34] based on only eight replicates. We used the round figure of 1000 in our estimates.

### DNA Extraction and Sequencing

We randomly picked three MA lines (MA31, MA47 and MA55) and grew them to a density of 1×10^7^ cells/ml in HL-5 media (10 g bactopeptone #2, 5 g yeast extract, 10 g glucose, 0.35 g Na2HPO4, 0.35 g KH2PO4, 1 ml 100x trace elements, pH 6.4–6.6). We collected cells from 100 ml culture and extracted good-quality DNA using a protocol developed by Mariko Katoh-Kurasawa as follows: we washed the cells three times with 50 ml of KK2 buffer by centrifuging (1300 rpm for 3 minutes at 4°C), discarding the supernatant, and re-suspending the cells in fresh KK2 buffer. After the last wash, we re-suspended the cells in 10 ml nucleus-isolation buffer (40 mM Tris, 1.5% Sucrose, 0.1 mM EDTA, 6 mM MgCl_2_, 40 mM KCl, 5 mM DTT, and 0.4% NP40-Alternative) to lyse the cells and extract the nuclei. After 10 minutes incubation on ice, we spun the cells at 8000 rpm for 5 minutes before carefully removing the supernatant and re-suspending the nuclei in EDTA to a final concentration of 100–200 mM with 450 µl of STE solution, 10 µl of 20% SDS, 25 µl ddH_2_O, and 10 µl of 10 mg/ml Proteinase K, and incubating the samples at 60 degrees for 60 minutes. We performed a phenol-chloroform extraction and ethanol precipitation purification to extract the DNA. We prepared the genomic DNA for sequencing using the Illumina library-preparation kit (DNA Sample Prep Kit # FC–102–1001). We prepared the libraries for MA31 and MA47 at the same time and the library for MA55 at a later date. We initially sequenced MA31 and MA47 on an Illumina GA1 sequencer with 36 cycles on two lanes for each genome. We processed the resulting reads using the Illumina GA pipeline 1.3. After initial analyses we decided to increase the coverage and number of MA lines so we sequenced one additional lane for both MA31 and MA47 and three for MA55. We sequenced these lanes with 51 cycles and processed the reads with the Illumina GA pipeline 1.4. All reads were single-end reads, regardless of their length. We used Firecrest for image processing and Bustard for base calling. We used the PIQA pipeline [48] to assess the quality of the reads and determined that the sequencing quality declined after 31 bps. Therefore, we trimmed all the reads, regardless of their initial length to a length of 31 bases before aligning them to the reference sequence (Eichinger et al. 2005) (retrieved from www.dictybase.org on May 13, 2009). All reads used in this project are available under accession number SRA056497.

### Alignment using Bowtie

We mapped the reads to the reference genome with zero or one mismatch per read using Bowtie [49] (-n1 -S –best –solexa1.3-quals with a seed length of 28 bp). Bowtie uses Burrows-Wheeler indexing, which results in fast alignments of reads to larger genomes. We used SAMtools [50] to create a pileup file (pileup -c -f). The pileup file contains information about how the reads align to a given site in the genome. Each row in the pileup file represents a position in the genome and lists the chromosome, position, reference sequence, consensus sequence, consensus quality, SNP quality (both Phred-scaled), the root mean square mapping quality, read base for each read that aligned to the site, and the alignment quality for each read base. We used the pileup command to compile a consensus sequence using a Bayesian model [50], [51]. We extended the pileup file to the full-genome length, which also included uncovered sites. In addition to the consensus sequence compiled by the pileup command, we used the information in the pileup file to determine what percentage of the reads called for a particular base, and compiled a second consensus sequence based on a majority rule: a consensus base was called if at least 50% of the reads agreed. If two bases were tied at 50% agreement each, we did not make a base call and considered the position uncovered. Because *D. discoideum* has a haploid genome, we did not have to account for the possibility of heterozygosity.

### Repeat Sequences and Uniquely Mappable Positions

To reduce problems caused by repeated sequences, we limited our analyses to uniquely mappable sites. To identify nucleotides that can be mapped uniquely with 31-bp reads, we used the reference sequence and created all possible 31-bp reads for both strands and aligned the ‘fake’ reads to the reference sequence with zero or one mismatch (seed length 28 bp) using Bowtie (-n1–best -sam). As for the real reads, we compiled a pileup file in SAMtools (pileup -c -f). The mapping options that we used in Bowtie allowed the reads to map to multiple places. As a result, exactly 62 reads covered each uniquely mappable position. Based on this self-mapping, we determined that 59% (20,337,176 bases) of the nuclear genome could be mapped uniquely with 31-bp reads.

### Identification of Mutated and Unchanged Sites in the Nuclear Genome

To calculate the mutation rate, we identified all apparent unique point mutations as well as all the sites where the reference nucleotide was confirmed (Figure 2), being careful to apply the same criteria to changed and unchanged sites. Candidate mutations were characterized as single nucleotide substitutions, where the majority base in an MA line differed from the reference base with at least five-reads coverage and 90% agreement. In addition, an accepted mutation had to be unique to one MA line, because mutations are rare events and it is very unlikely that the same mutation occurs multiple times independently. Candidate mutations that appear in more than one MA line are much more likely to be errors in the reference genome, differences between our ancestor clone and the reference genome, or mutations that occurred while we were growing up cells to start our 90 lines. Therefore, if a candidate mutation was the majority base in any of the other two lines, even with coverage of just one read, we excluded it. In order to avoid bias, we implemented a parallel rule for unchanged sites. We considered a nucleotide in one MA line unchanged if the majority base agreed with the reference base and if the reference base was confirmed (again with any non-zero level of coverage and the majority rule) in the other MA lines.

### Error Rate

To calculate the error rate, we used an approach similar to that of Keightley et al. [13], determining the fraction of reads covering a base that did not agree with the majority of the reads and calculating the average error rate for each line individually. We calculated the overall average error rate as the average of the three MA lines.

### Mutation Rate in the Mitochondrial Genome

To estimate the mitochondrial mutation rate, we used similar procedures but accounted for possible heteroplasmy, because a single *D. discoideum* cell contains multiple copies of the mitochondrial genome. Following Haag-Liautard et al. [7], we assumed that mutations in the mitochondria were maintained through mutation-drift balance and that the likelihood of fixation of a mutation was equal to its current frequency, so that each heteroplasmic mutation adds to the estimated mutation rate according to its frequency (see their equation 2). We identified sites that could be mapped uniquely (97% of the mitochondrial genome) and were covered by at least 400 reads, which is a tenth of the average coverage of the mitochondria.

As in the nuclear analysis, we assumed that mutations arise independently and therefore only accepted candidate mutations that were called in a single MA line. Mutations that occurred in more than one line above the cutoff minimum frequency were not included. To calculate the mutation rate, we used equation (2) in Haag-Liautard [7]. We summed all the frequencies of mutations in one line and divided the sum by the number of sites meeting the same criteria as the mutations (covered by at least 400 reads and uniquely mappable) and the number of generations (1000). We estimated the mutation rate for each line individually and calculated the average mutation rate. We calculated the Poisson confidence interval for 19 mutations.

We used two approaches to choose a cutoff frequency *f* for distinguishing true mutations (often heteroplasmic) from sequencing errors. In method one, we calculated mutation rate estimates using 10 values of *f* from 0.01 to 0.10 and looked for a minimum that reflected not accepting too many sequencing errors as mutations and not missing too many real, low-frequency mutations. The estimates were in a relatively narrow range with the minimum at a cutoff of *f* = 0.03. Second, we used our empirical estimate of alignment/sequencing error for nuclear sites (see Results) to assess how likely we were, at each possible cutoff, to have sequencing errors leading to the acceptance of a false mutation. Our empirical estimate was in the range of 0.002 per read, and we doubled that to account for the fact that some sites were likely to be systematically more error-prone. At that error rate, we asked: if we have 400 reads, how low can we make the cutoff frequency *f* to keep the binomial probability of having more than 400×*f* erroneous reads below 1 across all our sites (i.e., lower than 6.7×10^−6^ for each of the 150000 mitochondrial sites in the three genomes)? That answer also yielded a cutoff of *f* = 0.03, so we used that for our reported estimate.

### Other Mapping Tools

In addition to the results presented here, we performed several variations of the analysis using: 36-bp reads, a different mapping tool, different ways to compile a consensus sequence, varying filtering parameters, and a ML method, respectively. We initially decided to cut all our reads to 31-bp because of the declining quality of the reads after base 32. To align the 36-bp reads, we used the same approach as described above. We also used a different mapping tool, MAQ, to align the reads and compile the majority consensus sequence. To identify mutations, we compiled a consensus sequence based on the majority call of all the reads aligning to one position. We used this consensus sequence to estimate our mutation rate. We also performed the same analysis using the consensus sequence compiled by MAQ or SAMtools (ambiguous calls were replaced by the majority base call). Using the consensus sequence consistently resulted in a large number of candidate mutations that were later identified as false positives by Sanger sequencing. We performed all of these analyses with varying filtering parameters. We tried the ML method developed by Lynch [9], [35] to estimate the mutation rates. The low number of mutations in our data set, however, did not work with this method; after two iterations, all the mutations were exhausted and the program was unable to estimate the mutation rate.

### Confirmation of the SNPs using Sanger Sequencing Technology

We used Sanger sequencing to confirm the computationally predicted unique mutation in MA31 by sequencing all MA lines and the ancestor (using forward primer: 5′-CTTTCAAGGTGAAGCGAATAAAACA and reverse primer: 5′-ACATATGCTTTGAGTGGGAGATTAC).
